# Reliability of Blue-Emitting Eu^2+^-Doped Phosphors for Laser-Lighting Applications

**DOI:** 10.3390/ma11091552

**Published:** 2018-08-28

**Authors:** Matteo Buffolo, Carlo De Santi, Marco Albertini, Donatella Carbonera, Gian Andrea Rizzi, Gaetano Granozzi, Gaudenzio Meneghesso, Enrico Zanoni, Matteo Meneghini

**Affiliations:** 1 Department of Information Engineering, University of Padova, via Gradenigo 6/b, 35131 Padova, Italy; carlo.desanti@dei.unipd.it (C.D.S.); gauss@dei.unipd.it (G.M.); zanoni@dei.unipd.it (E.Z.); matteo.meneghini@dei.unipd.it (M.M.); 2 Department of Chemical Sciences, University of Padova, via Marzolo 1, 35131 Padova, Italy; albertini.marco.87@gmail.com (M.A.); donatella.carbonera@unipd.it (D.C.); gianandrea.rizzi@unipd.it (G.A.R.); gaetano.granozzi@unipd.it (G.G.)

**Keywords:** phosphors, laser-lighting, degradation, europium

## Abstract

This paper investigates the reliability of blue-emitting phosphors for Near-UV (NUV) laser excitation. By means of a series of thermal stress experiments, and of stress under high levels of optical excitation, we have been able to identify the physical process responsible for the degradation of Eu^2+^-activated alkaline-earth halophosphate phosphors under typical and extreme operating conditions. In particular, for temperatures equal to or greater than 450 °C the material exhibited a time-dependent drop in the Photo-Luminescence (PL), which was attributed to the thermally induced ionization of the Eu^2+^ optically active centers. Several analytical techniques, including spatially and spectrally resolved PL, Electron Paramagnetic Resonance (EPR) and X-ray Photo-emission Spectroscopy (XPS) were used to support this hypothesis and to gain insight on the degradation process. By means of further tests, evidence of this degradation process was also found on samples stressed under a relatively low power density of 3 W/mm^2^ at 405 nm. This indicated that the optically (and thermally) induced ionization of the optically active species is the most critical degradation process for this family of phosphorescent material. The operating limits of a second-generation Eu-doped halophosphate phosphor were also investigated by means of short-term stress under optical excitation. The experimental data showed that a threshold excitation intensity for continuous pumping exists. Above this threshold, decay of the steady-state PL performance and non-recoverable degradation of the material were found to take place. This behavior is a consequence of the extremely harsh excitation regime, mainly due to the thermal management capabilities of the substrate material employed for our experimental purposes rather than from intrinsic properties of the phosphors.

## 1. Introduction

The generation of white light by means of phosphor-converted Light-Emitting Diodes (LEDs) based on a blue-emitting Gallium Nitride chip is the common approach adopted by modern Solid-State Lighting (SSL) solutions to achieve reliable and efficient light sources for a wide range of general lighting applications. However, alternative SSL solutions based on laser diodes instead of LEDs are starting to emerge. Compared to LEDs, laser diodes offer two main advantages: an increased efficiency at high driving current densities, i.e., for high optical power single source operation, and a simplified and more effective design of the optical system. The first point is related to a reduced effect of the so-called efficiency droop phenomenon in Laser Diodes (LDs). The term efficiency droop describes the set of physical mechanisms responsible for the decrease in internal quantum efficiency of the device as the injected current increases. This process, typical for LEDs, is usually ascribed to Auger recombination [1] or carrier spill-over [2]. On the other hand, in LDs operated above threshold the emission of coherent light is associated with the stimulated recombination of carriers: compared to the spontaneous emission process responsible for photon emission in LEDs, this is a much faster process, less affected by the formerly cited loss mechanisms.

Efficiency droop ultimately limits the amount of optical power attainable from an LED for a given chip area, meaning that if high-brightness high-efficiency operation is to be achieved, the area of the semiconductor chip needs to be increased. However, not only this approach would increase the manufacturing costs, but it would also worsen the optical performance of the lighting system in terms of light collimation. In an optical system, the product of a source emitting area and the solid angle of the emitted beam, called *etendue*, does not decrease if the optical power is conserved. This means that the collimation of a (relatively) large area source (up to 3–4 mm^2^ for a modern high-power LED) featuring a large-aperture Lambertian emission pattern, would require an increase in the dimensions of the optical elements, thus increasing the complexity and the cost of the final illuminator. On the other hand, laser diodes feature a very small emitting area, in the orders of tens of square microns, which allows for the design of cheaper low-divergence LD-based light sources, such as the ones employed in digital laser projectors.

Laser-Activated Remote Phosphors (LARP) lighting systems rely on two main white-light generation approaches. These are either based on the partial conversion of blue light (450 nm–460 nm) through a yellow phosphor blend, or on the total phosphor conversion of NUV light (380 nm–410 nm) into blue light, which is then partially converted to longer wavelengths to attain the desired chromaticity point. In principle, assuming phosphors and LDs with comparable efficiencies, the first approach shows an intrinsic advantage due to the reduced energy loss in the phosphor conversion process (about 12.5%, considering excitation wavelengths of 400 nm and 450 nm). However, for high-power operation, the efficiency of the light source at high current densities is the dominant factor in determining the global efficiency of the LARP system. With regard to LEDs, this translates into a clear supremacy of NUV sources over conventional blue emitters [3], determined by the more prominent efficiency droop of longer wavelength devices at high injection currents. However, for LDs operating in stimulated emission regime the intrinsic inefficiency of blue emitters is less pronounced, and the choice of a specific lighting approach becomes more bound to the technological limitations of state-of-the-art devices and to the optical design of the illuminator rather than to the physical principles ruling the emission processes.

This work focuses on the reliability of Eu-doped halophosphate blue-emitting phosphors for NUV (405 nm) semiconductor laser excitation. Devices featuring such emission wavelength have already proven their reliability and the capability for continuous operation at output power in excess of 7 W [4]. The high light intensity attainable by those devices requires phosphor morphologies with high thermal conductivity, to deal with the self-heating due to Stokes loss and to the non-unitary quantum efficiency, as well as an elevated chemical stability under high optical intensity excitation and high-temperature environment. Under such operating conditions several degradation processes can limit the lifetime of Eu-doped phosphors. High-temperature treatment in an oxygen-rich environment can severely reduce the optical efficiency of the material by inducing the oxidation of the Eu^2+^ centers [5,6,7,8], of the host lattice [8] or of the cations present in correspondence of the surface of the host material [9], as well as the migration of the optically active centers towards different crystallographic sites [10]. On the other hand, the exposure to high-intensity and high-energy UV light can induce the formation of traps in the host lattice [11,12,13,14], the photo-ionization of the Eu^2+^ ions [13,15,16] or their migration towards the cation layer of the host lattice [17]. The operating temperature, the external environment, as well as the wavelength and the intensity of the optical excitation are all important factors that contribute in determining the dominant degradation process for a specific family of phosphorescent materials. Therefore, aim of this work is to analyze the variation in the luminescence and in the chemical and morphological properties of Eu-doped halophosphate blue-emitting phosphors under different operating regimes. To pinpoint the root causes of degradation, and to determine the optical excitation bounds for safe Continuous Wave (CW) operation of the phosphors, several thermal and optical accelerated stress tests were performed. The experimental results showed that rapid and non-recoverable degradation of the PL properties of the material occurs once a specific excitation threshold is reached. This kind of degradation could be ascribed to the thermally and optically induced ionization of the Eu^2+^ centers into Eu^3+^ ions. The details on sample preparation and characterization, and regarding the investigation on the aforementioned degradation process are reported in the following paragraphs.

## 2. Materials and Methods

### 2.1. Material under Analysis

**Figure 1:**
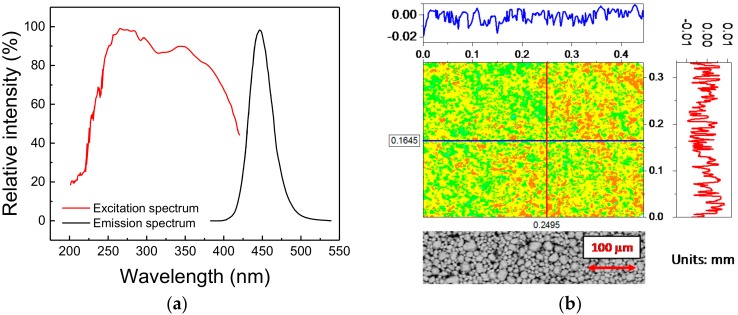
(**a**) Emission and excitation spectra of the phosphor under investigation, as provided by the manufacturer. (**b**) Height profile map (top) and back-scattered electrons Environmental Scanning Electron Microscopy (ESEM) image (bottom) of a portion of the surface of the deposited material.

The phosphorescent material under investigation is a commercial blue-emitting pigment developed to attain a peak emission wavelength of 448 nm (blue), whereas the excitation range, tuned for UV light sources, ranges from 200 nm to 400 nm, with characteristic peak excitation wavelengths at 254 nm and 365 nm (Figure 1a). The material belongs to the family of Eu^2+^-activated alkaline-earth halophosphates, usually employed in the past as luminescence materials for fluorescence lamps, and more recently for SSL [18]. The basic chemical composition of this material is given by

(1) $$\;{\mathrm{Sr}}_{5}{{(\mathrm{PO}}_{4})}_{3}{\mathrm{Cl}\;:\mathrm{Eu}}^{2+}$$

where, in principle, other alkaline-earth metal ions such as Ca or Ba can be employed in conjunction with Sr to tune, within specific limits, both the emission and excitation spectra, as well as to optimize the conversion efficiency of the material and its reliability.

As to the microstructure, the phosphorescent material is a white powder, whose typical grain size ranges from 5 µm to 40 µm, with an average diameter of about 16 µm. For experimental purposes, the powder was deposited onto a thermally conductive sapphire substrate (1/2 inch in diameter) by a low-rpm spin-coating technique. To this aim, the powder was mixed in a 50:50 ratio with benzyl alcohol, employed as a carrier fluid. A fixed amount of mixture was then spin-coated on top of the substrate. Finally, the phosphor-covered substrate was placed inside a thermal chamber at 250 °C for 7 min, to let the solvent evaporate. After the deposition procedure, the phosphor powder is spread in a solid phase across the surface of the substrate: since no foreign materials are present in the sample after the evaporation of the alcohol, this procedure ensures that only the luminescent material is deposited and characterized. Despite the absence of an encapsulant may increase the risk of contamination and degradation by external agents (moisture, oxygen, dust, etc.), the possibility of analyzing only the bare material offers far more advantages.

The morphological quality of the deposition was evaluated by means of a profilometer with scanning red laser interferometer (model MSA-500 from Polytec, Waldbronn, Germany). As highlighted by Figure 1b, the variation in the height of the deposition, which shows a peak-to-peak distance of 70 µm with a variance of 5.3 µm, is compatible with the dimensions of the phosphor grains: this suggests that a very good level of deposition quality could be achieved with the adopted technique. That conclusion was further demonstrated by the Environmental Scanning Electron Microscopy (ESEM) (model Quanta 200 from FEI, Hillsboro, OR, USA)) images taken on an untreated sample, here reported at the bottom of Figure 1b: the detected variations are mostly related to the different particles dimensions rather than to a non-uniform deposition.

**Figure 2:**
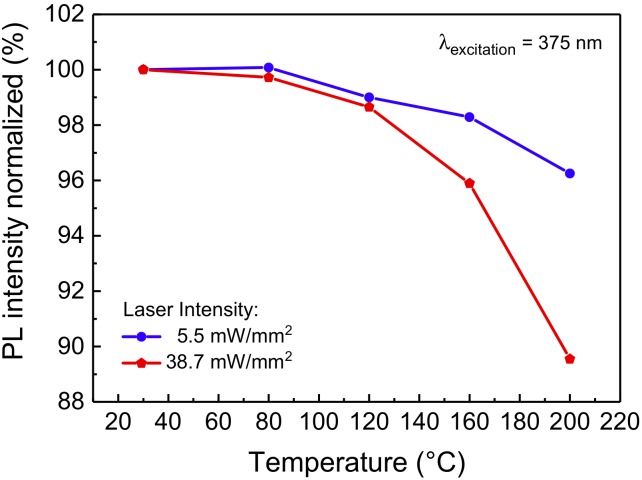
PL emission of the blue-emitting phosphor, normalized to the respective value at 30 °C for each excitation intensity, as a function of the substrate temperature. The optical source employed for this measurement is a solid-state 375 nm laser (model LBX-375, Oxxius, Lannion, France).

With regard to its optical performance, the material exhibits good thermal quenching behavior at low-intensity CW 375 nm excitation, showing only a moderate 4% decrease when increasing the sample temperature from 30 °C to 200 °C (Figure 2): such behavior was found to be comparable to the reported thermal quenching behavior of blue-emitting phosphors belonging to the same family [19,20]. At excitation intensity higher than 5.5 mW/mm^2^, a higher drop in PL efficiency is observed, possibly due to the increased self-heating of the material.

### 2.2. Experimental Details

**Figure 3:**
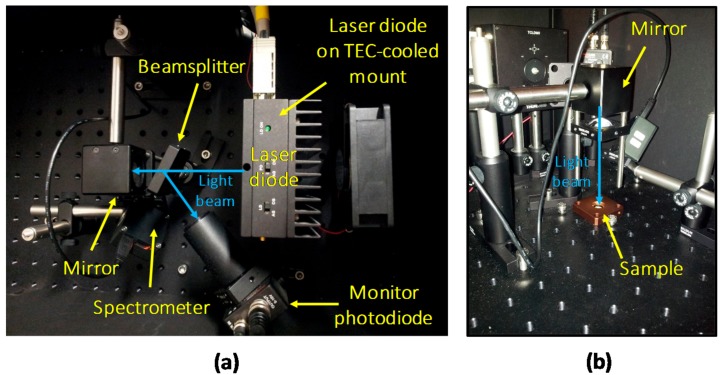
Experimental setup for optical stress and characterization under 405 nm LD excitation: top (**a**) and side (**b**) views.

To perform optical stress and characterization of the deposited material, a custom setup for Photo-Luminescence (PL) measurement was designed (Figure 3). A thermo-controlled high-power 405 nm LD, capable of generating more than 2 W at a drive current of 1.3 A, was employed as light source. The LD was operated in constant optical power mode by maintaining constant the reading of a monitoring photodiode (PD), on which part of the emitted light was redirected by means of a beam-sampler (model BSF10-A from Thorlabs, Newton, NJ, USA). The main collimated light beam exiting from the beam-sampler was then reflected with an angled 45° mirror, and focused onto the horizontally lying sample with a suitable focusing lens (from Thorlabs).

In order to obtain a specific optical excitation density, both the power and the spatial distribution of the excitation beam must be measured. Regarding the former, a complete optical calibration of the setup was carried out by mapping the reading of the feedback photodiode with the measurements of a factory-calibrated power-meter. The extension of the excitation spot was measured by evaluating with a Dino-Lite digital microscope (Anmo Electronics Corporation, Taipei, Taiwan) the area (at Full Width Half Maximum) of the emission spot with the LD driven above threshold. Finally, the two measured values were employed to compute the excitation density, in W/mm^2^, of the light beam.

The surface chemical composition of the samples was investigated by XPS using a custom equipment working at a base pressure of 10^−10^ mbar and adopting an EA 125 Omicron electron analyzer (Scienta Omicron, Taunusstein, Germany) with a five channeltron detector. The XPS data were collected at room temperature using the Al K_α_ line (hv = 1486.6 eV) of a non-monochromatized dual-anode DAR400 X-ray source. High resolution spectra were acquired using 0.1 eV energy steps, and 20 eV pass energy. The multi-peak analysis of Eu 3d photo-emission lines was performed by means of Voigt function and subtracting a Tougaard background [21]. The binding energy (BE) scale was calibrated with respect to the C1s signal due to adventitious carbon contamination on sample surfaces, assuming a binding energy of 285.0 eV. All samples presented a strong charging effect (the material is not conducting) of about 30 eV.

Electron spin properties of the Eu centers were investigated by electron paramagnetic resonance (EPR) spectroscopy. All the measurements were performed on an X-Band Bruker Elexsys E580 spectrometer equipped with an ER4116DM dual mode resonator (both from Bruker Corporation, Billerica, MA, USA) operated in its perpendicular mode (ν = 9.815 GHz). EPR spectra were recorded at room temperature applying a 10,000 G wide magnetic field sweep centered at 5050 G; a 100 kHz modulating field of 3 G amplitude was applied to achieve proper phase sensitive detection; microwave power was set to 4.697 mW; 8192 data points per spectra were collected, resulting in a 335.5 s sweep time.

## 3. Experimental Results

### 3.1. Effects of Thermal Stress

**Figure 4:**
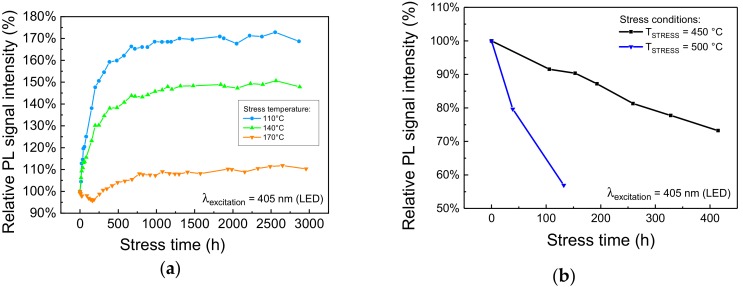
Trend of the PL spectra, integrated from 450 nm to 460 nm, measured during (**a**) moderate and (**b**) high-temperature treatment in air.

Due to the Stokes shift, and to the non-unitary quantum yield, during high optical intensity operation the luminescent material can reach very high temperatures that can negatively affect both the optical performance of the material, by lowering its PL efficiency, and its long-term reliability, by inducing material degradation through temperature-activated degradation processes [5,7]. Even though we previously demonstrated that this kind of phosphorescent material starts becoming limited by thermal quenching for temperatures higher than 200 °C (Figure 2), a comprehensive analysis on the effects of thermal aging is due to identify the limiting operating conditions in LARP luminaires with reduced thermal management capabilities. To this aim, a series of nominally identical phosphor samples were submitted to extended thermal cycles in temperature-controlled climate chambers. The characterization of the samples, performed at regular intervals, was carried out by means of low-intensity transmission PL measurements. The results of the long-term aging experiments at constant temperature are summarized in Figure 4.

The degradation kinetics show no tangible worsening of the PL emission for stress temperatures lower than 170 °C (Figure 4a). By contrast, the material under investigation exhibited a noticeable increase in luminescence. This process is possibly caused by the annealing of the material and did not induce significant changes in the spectral shape of the emitted light, and its time constant was found to be not thermally activated. On the other hand, the degradation kinetics at high (≥450 °C) stress temperature, reported in Figure 4b, show remarkable PL signal degradation, even during the first 50–100 h of stress. In this case, the degradation process was found to be thermally activated, with an activation energy for the Time-To-Failure at 75% (TTF_75%_), the time required by the sample to lose 25% of its original PL signal strength, around 1.6 eV. As will be shown in the following paragraphs, this behavior may be explained by the thermally induced ionization of Eu^2+^ centers due to high-temperature baking in air environment [8], or as a consequence of optical stress under high-intensity radiation [16].

### 3.2. Stress Under Optical Excitation

As described previously, for high optical power density stresses a 405 nm high-power laser diode was employed as optical source. Due to the easy availability and the high efficiency of modern 405 nm solid-state sources, this excitation condition represents the sweet spot from an engineering point of view [3]. However, the reduced conversion efficiency of the phosphorescent material at this lower wavelength may pose various reliability issues, especially related to the increased self-heating of the material. To investigate the degradation processes induced by near-UV optical excitation, we started our analysis by submitting a phosphor sample to a 140 h long stress at 3.2 W/mm^2^, with an ambient temperature of 25 °C.

**Figure 5:**
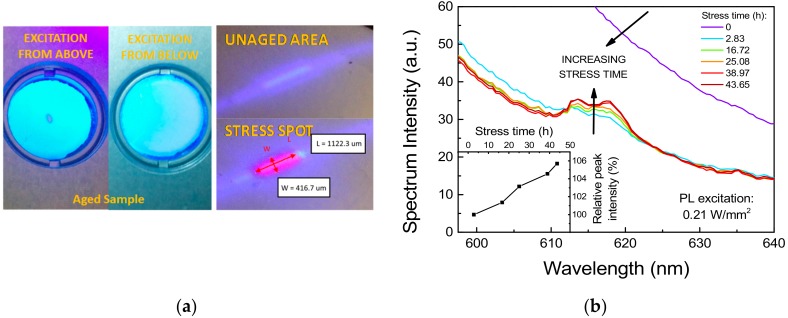
Stress under 405 nm excitation at 3.2 W/mm^2^, T_AMB_ = 25 °C. (**a**) Image of the photo-luminescence from the first excitation spot after 140 h of stress. On the left: emission from top and bottom surfaces with 405 nm LED excitation. On the right: comparison of the PL emission from the stress spot and from a portion of untreated surface (red markings highlight the approximate dimension of the red-emissive spot). (**b**) Trend during optical stress on a second spot of the PL spectra measured at very-low intensity (I_exct_ ≈ 0.21 W/mm^2^). Inset graph shows the trend of the red luminescence over stress time.

As shown in Figure 5a, the prolonged exposure to high-intensity radiation induced a complete extinction of the PL emission in correspondence of the excitation spot. Interestingly, the non-emissive spot could only be observed by direct excitation of the phosphor surface, whereas upward illumination induced a uniform photo-emission. Considering that no complete absorption of the excitation radiation was taking place, and thus the upper portion of the material could still be partially pumped from the back, this behavior may suggest that only the upper layers of phosphors were affected by degradation. In addition to that, a reddish PL signal, absent in untreated areas of the sample, could be detected by low-intensity (0.21 W/mm^2^) 405 nm laser excitation of the stressed spot (Figure 5a).

To investigate step-by-step the spectral changes in the long wavelength region and to acquire further details on the kinetics of this degradation phenomenon, the same stress experiment was repeated on a second spot of the same phosphor sample, while recording the low-intensity PL spectrum at regular stress intervals. As reported in Figure 5b, the intensity of the red luminescence peak was found to gradually increase with stress time in an almost linear fashion. Interestingly, the spurious red luminescence was found to have an emission linewidth ranging from about 609 nm to 624 nm, compatible with the characteristic peak emission wavelength of Eu^3+^-related optically active centers [22].

**Figure 6:**
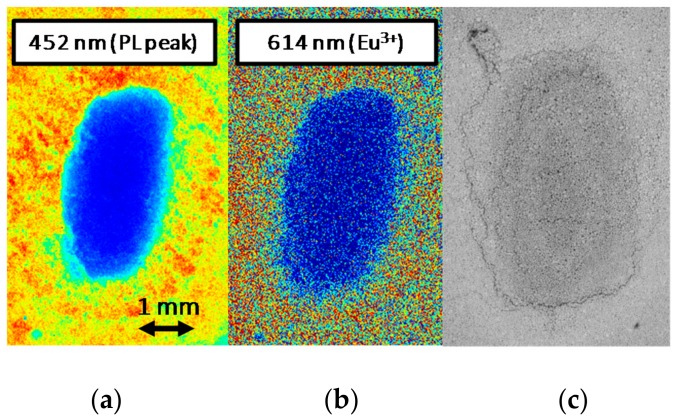
Analysis of the degraded phosphor surface after stress under 405 nm, 3.2 W/mm^2^ excitation at T_AMB_ = 25 °C. Spatially and spectrally resolved PL map of the stressed spot measured at low-intensity 405 nm laser excitation with selective filtering at 452 nm (**a**) and 614 nm (**b**); back-scattered electrons ESEM imaging of the degraded surface (**c**).

The extension of the degraded red-emitting area was then evaluated by means of spatially and spectrally resolved PL measurements carried out with 405 nm light excitation (Figure 6). By means of a scientific Electron Multiplying Charge-Coupled Device (EMCCD) camera, model Luca S DL-658M-TIL by Andor (Abington on Thames, UK), paired with a VariSpec tunable liquid crystal filter manufactured by CRI (Waltham, MA, USA), we were able to selectively map the spatial emission from the surface of material at determined wavelengths within the 400–720 nm range. The comparison between the monochromatic emission maps at 452 nm and 614 nm, respectively the peak emission wavelengths of the phosphors and of the red luminescence, showed no peculiar differences. In these hot-cold color-coded images, both measurements showed complete annihilation of the PL in correspondence of the center of the emission spot. Assuming from optical images that the red emission originates from the center of stress spot, this may indicate that under the low-intensity LED-driven excitation employed for the measurement, the process responsible for the observed spurious emission is relatively weak. This reduced emission rate may either be due to a low concentration of the chemical species that assist the spurious red emission process, or to an inherently low efficiency of the process itself, possibly related to a non-optimized chemical configuration of the host lattice for this lower-energy optical transition.

Finally, back-scattered electrons ESEM imaging of the degraded excitation spot revealed stress-induced changes both in the morphology and in the chemical composition of the sample area subjected to high optical excitation (Figure 6). This latter consideration supports the hypothesis that the localized annihilation of the PL signal observed after moderate optical stress may be related to a variation in the chemical properties of the material.

### 3.3. Physico-Chemical Analysis of the Phosphors Samples

To identify the physical mechanisms responsible for the optically induced degradation of the material, we performed further physico-chemical analyses on the treated phosphors samples. The drop in the PL signal could be related to an increase in a characteristic red emission around 613 nm, possibly ascribed to the photo-and/or thermally induced ionization of the Eu^2+^ centers into Eu^3+^ ions. Therefore, we further investigated this hypothesis by analyzing several samples of thermally and optically stressed phosphors by means of various material analysis techniques, including high-sensitivity optical spectroscopy, EPR and XPS.

#### 3.3.1. High-Sensitivity Optical Spectroscopy

**Figure 7:**
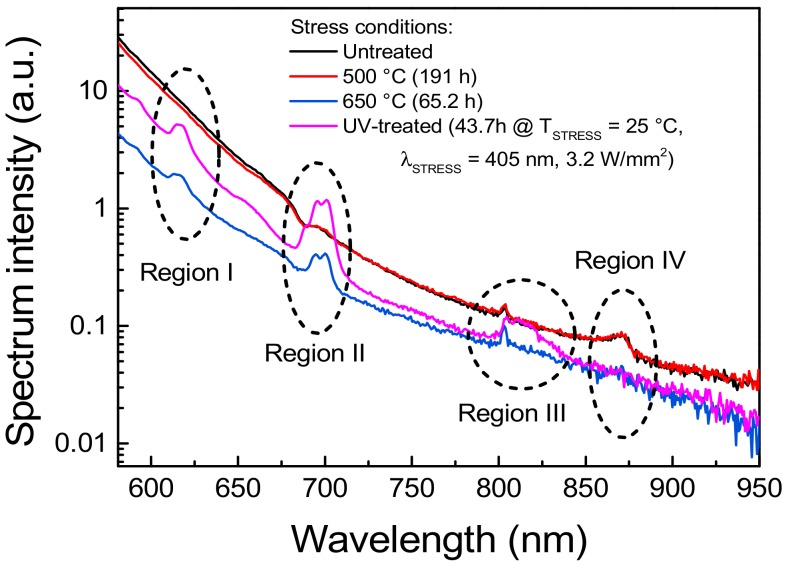
Normalized low excitation intensity PL spectra in the long wavelength region recorded by means of a high-sensitivity spectrometer. The UV treatment referred to in the plot legend corresponded to the 405 nm, 3.2 W/mm^2^, T_AMB_ = 25 °C stress described in Figure 5b.

By means of a high-sensitivity spectrometer, model CAS 140CT by Instrument Systems (Munich, Germany), the emission spectrum of the luminescent material was further investigated. The results of this analysis, reported in Figure 7, show that because of the thermal and/or optical stress, characteristic spectral lines become visible or disappear, depending on the wavelength region. In region I, UV treatment and annealing at 650 °C in air induced the increase in the emission centered around 615 nm, which is commonly ascribed to the D50→F72 Eu^3+^ transition [6,22]. Similarly, in region II the same stress procedure generated a spurious emission peak around 695 nm and 705 nm, which can be related to the Eu^3+^ D50→F74 transitions [22,23]. On the other hand, the spectral line highlighted in region III around 800 nm, detected also on the untreated sample, was not affected by either thermal of UV stress. Considering that this emission corresponds to the D50→F76 transition of the Eu^3+^ compounds, we can suppose that this characteristic transition is related to the few Eu^3+^ centers that are generated as a consequence of the manufacturing process, and that the adopted stress conditions did not significantly contribute in increasing the concentration of the associated emissive sites. Finally, despite the luminescence peak appearing in region IV around 870 nm could not be associated with any of the Eu^2+^ or Eu^3+^ characteristic spectral lines, its reduced intensity after high temperature or UV stress suggests a possible correlation with the former of the two species. These experimental results support the hypothesis that high levels of optical excitation, as well as high-temperature stress, can induce degradation of the material due to the ionization of the optically active Eu^2+^ centers into Eu^3+^ ions.

#### 3.3.2. Results of EPR Analysis

**Figure 8:**
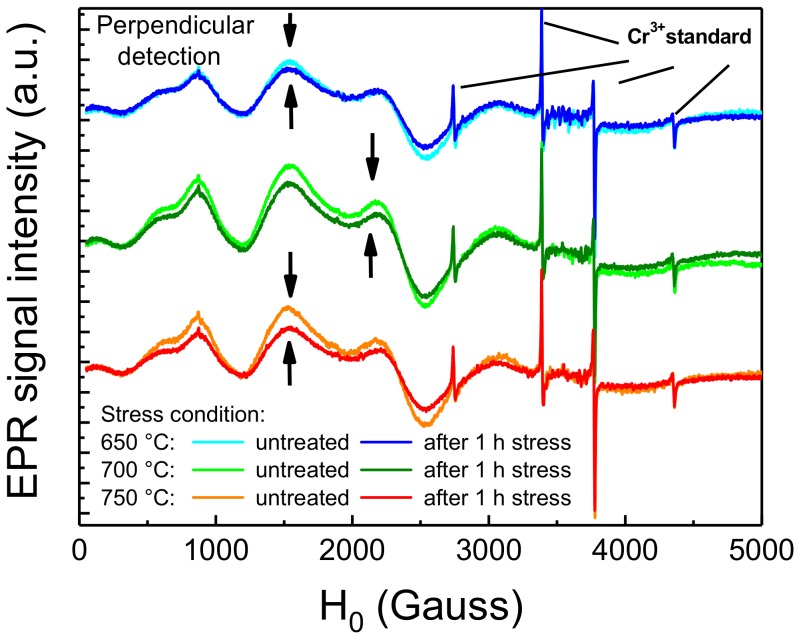
Room-temperature EPR signal of Eu^2+^ centers before and after 1 h thermal stress at 650 °C to 750 °C.

The hypothesis formulated in the previous paragraph was also supported by EPR spectroscopy on thermally treated samples. EPR spectroscopy is a material analysis technique aimed at investigating atoms, ions or molecules with unpaired valence electrons. This technique exploits the effect of an external magnetic field to separate the spin levels and of a microwave electromagnetic field to promote spin transitions. The resonant frequency depends on the surrounding environment of the paramagnetic center and give information in term of concentration, bonds, active nuclei and coordination sphere. Since in the material only the optically active Eu^2+^ centers, and not the Eu^3+^ species resulting from its degradation, feature an electronic structure with unpaired valence electrons in the 4f^7^ orbital, this technique proved to be suitable for our investigation. To ensure repeatability of the measurements, the material was stressed and measured in situ inside the EPR tube, a quartz-made tube-shaped holder that is inserted inside the resonant EPR cavity, suitable for probing solid and liquid-phase samples. Moreover, reference and post-stress measurements were both performed. To this aim, the degradation process was accelerated by performing 1 h long thermal stresses at the temperatures of 650 °C, 700 °C and 750 °C. Fluctuations of the EPR signal, due to instrumental sources, were considered by normalizing the spectra to the four sharp signals of a reference (solid) Cr^3+^ standard, introduced inside the tube prior to each measurement and then removed. The results of the EPR analyses are reported in Figure 8. As a consequence, to the thermal stress, the EPR signals within the 0–3000 Gauss region arising from the Eu^2+^ centers experienced a decrease in intensity, showing greater relative decrease for higher stress temperatures. This indicates that very high temperature short-term stress can induce a significant decrease in the concentration of the Eu^2+^ species within the phosphor. This thermally induced decrease was also found to be compatible with previous reports on the degradation of Eu-doped phosphors submitted to high-temperature thermal aging [7].

#### 3.3.3. Results of XPS Analysis

**Figure 9:**
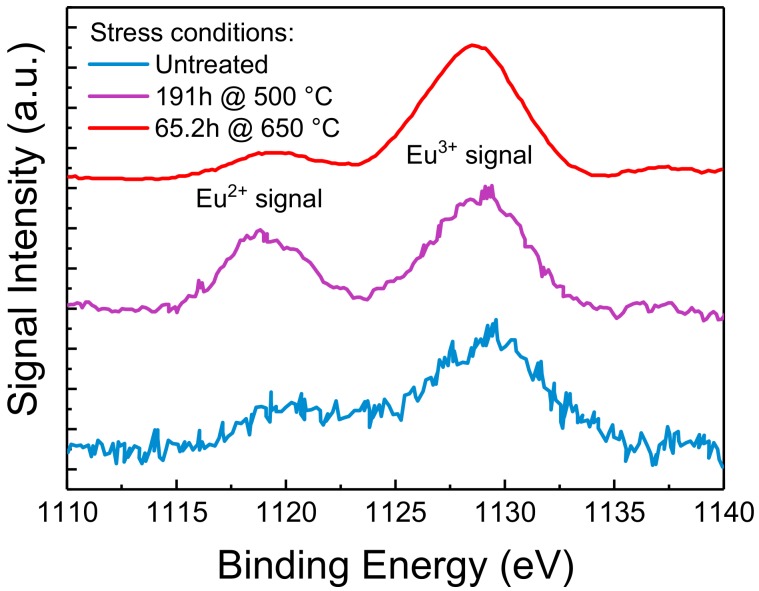
XPS data of the Eu 3d_5/2_ core level of untreated and thermally treated Eu-doped phosphors. The spectra have been plotted after a Tougaard background subtraction [21], a normalization between their minimum and maximum values, and shifted vertically to ease readability. Based on the adopted measuring conditions, the average information depth is of few nm.

We have previously showed how moderate optical stress only degrades the surface layers of the phosphorescent material. To further understand the effects of this localized degradation on the chemical composition of the material, we characterized both treated and untreated samples by means of XPS, a surface analysis technique capable of quantitatively evaluate the chemical composition and the electronic state of the elements within the first nanometers of the material under investigation. The results are reported in Figure 9.

In XPS spectra, the signal intensity, i.e., the number of collected photo-emitted electrons, is proportional to the amount of a specific element inside the probing volume, whereas peaks in correspondence of specific BEs identify the electron configuration of the atoms in the material under investigation. With XPS being a quantitative analysis technique, typically to the parts per thousand range, the experimental data suggest that thermal stress induced a variation in the amount of Eu centers near the surface of the material. In particular, looking at the Eu 3d core levels, two different valence states (+2 and +3) of Eu ions can be observed. Each set exhibits simple spin-orbit doublet peaks, which split off 30 eV from each other. In Figure 9 we report only the lower BE Eu 3d_5/2_ components: the peaks located at lower BE can be assigned to Eu^2+^ and the higher BE one to Eu^3+^. In particular, thermal aging at 500 °C showed a comparable increase of both the Eu^2+^ and Eu^3+^ XPS peaks, suggesting a thermally driven diffusion of the two chemical species towards the surface. This phenomenon is quite common and is referred as thermal driven surface segregation. On the other hand, stress at 650 °C also induced an increase in the relative amount of Eu^3+^ with respect to the concentration of Eu^2+^: this observation is compatible with the oxidation of Eu^2+^, assisted by high-temperature and by the oxygen-rich environment near the surface of the sample [6]. A similar relative increase in Eu^3+^ was previously observed in literature by means of XPS on both thermally and UV-treated phosphors, as reported in [7,24]. These results confirm once again the role of oxidation of the Eu^2+^ centers in the degradation of the material under investigation.

## 4. Operating Limits under Optical Excitation

In the previous paragraph we showed that Eu-doped blue-emitting phosphor subjected to moderate levels of optical stress can degrade due to irreversible ionization of the of Eu^2+^ centers. From an engineering point of view, it is important to identify the limits for continuous excitation of the material, above which consistent PL efficiency decay or material degradation occurs. To this aim, an optical step-stress experiment was carried out on a second-generation Eu-doped halophosphate phosphor, sharing with the previously investigated material the (general) chemical composition and the behavior under optical excitation. A specific surface spot was submitted to 12 s long stress steps under increasing 405 nm optical excitation levels, from 0.5 W/mm^2^ to 3.5 W/mm^2^. A reference measurement at 0.5 W/mm^2^ was taken before and after each stress step to discriminate between thermal quenching-induced PL decay and non-recoverable phosphor degradation. A cool-down period of 300 s was employed before low-intensity characterization to let the sapphire substrate and the phosphors dissipate the heat accumulated during the stress. Finally, with the aim of attaining high temporal resolution during the acquisition of the PL signal, we employed as light detector an amplified photodiode (model PDA36A-EC from Thorlabs), carefully shielded from the 405 nm laser light reflected from the sample and connected to an oscilloscope.

**Figure 10:**
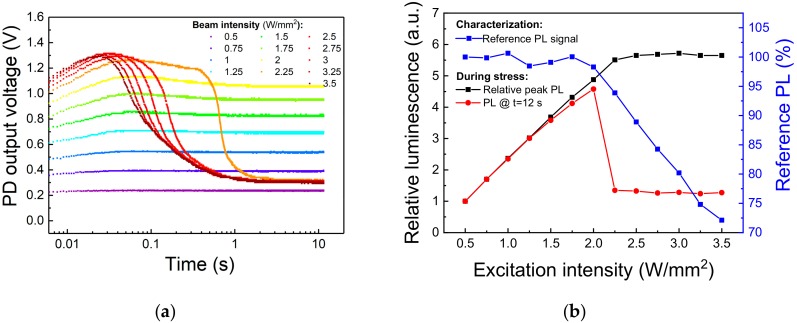
Optical step-stress under 405 nm excitation at T_AMB_ = 25 °C. (**a**) Trends of the PL signal under increasing values of optical excitation. (**b**) Variation of the reference (low-intensity) and peak PL signals in function of the stress excitation intensity.

The experimental results, reported in Figure 10, show that after an initial PL increase related to the turn-on transient of the excitation source, a sudden PL decay occurred after about 400 ms of stress at 2.25 W/mm^2^. Above this excitation intensity, the steady-state PL signal, i.e., the PL at the end of the 12 s stress step, drops to a fixed value corresponding roughly to the emission during 0.5 W/mm^2^ stress, whereas the reference PL measurements starts decreasing in amplitude, meaning that stress above 2.25 W/mm^2^ induced permanent degradation to the phosphor (Figure 10b). In particular, we can see how above this excitation intensity the delay between the beginning of the stress and the rapid PL decay decreases with increasing light intensity. This behavior can be explained by considering that above a certain (power-dissipation) threshold, the self-heating of the material reduces the rate of optical emission, increasing even more the quantity of incident energy converted into heat. This positive feedback rapidly increases the temperature of the stress spot, thus annihilating the emission from this area and inducing permanent degradation to the phosphor particles located nearby. When this critical stress intensity is reached, the PL becomes more dependent on the phosphorescent material surrounding the excitation spot rather than on the severely heated (and partially degraded) excitation spot itself, as testified by the constant value of the PL emission at the end of the stress for excitation intensities greater than the threshold value (Figure 10b).

**Figure 11:**
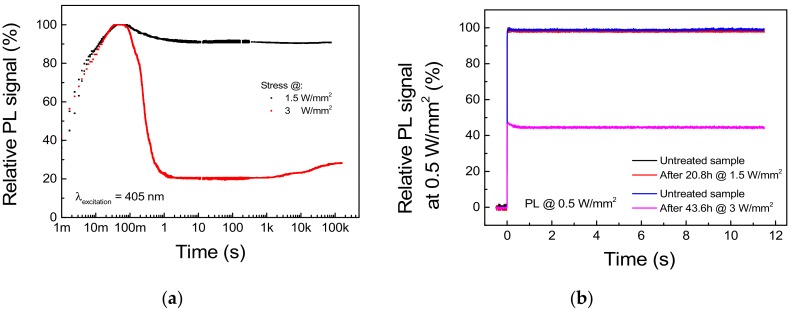
Stress under 405 nm excitation at 1.5 and 3 W/mm^2^, T_AMB_ = 25 °C. (**a**) PL trends during stress. (**b**) Reference PL signal measured at 0.5 W/mm^2^ before and after each stress cycle.

To prove that the excitation threshold I_TH_ previously found represents a bound for safe CW excitation of the phosphor, we carried out two long-term stresses under optical excitation levels of 1.5 W/mm^2^ and 3 W/mm^2^, respectively below and above I_TH_. The PL transient was registered from the very beginning of the experiments by means of the same setup describe above. Moreover, to discriminate between thermal quenching-induced PL decay and non-recoverable phosphor degradation, a reference PL measure at a safe 0.5 W/mm^2^ excitation level was performed before and after the stress. The results of the experiments are reported in Figure 11.

Regarding the stress under 1.5 W/mm^2^ optical excitation, the waveform of the PL signal acquired during stress and reported in Figure 11a shows a 10.5% difference between the peak and the steady-state value, whereas only a <0.8% decay in the reference PL measurement was registered (Figure 11b). This suggests that the PL decay experienced by the sample is mostly related to a thermal quenching phenomenon and that 1.5 W/mm^2^ represents a safe pumping intensity for the given deposition conditions and the thermal management capabilities of the system. On the other hand, stress at 3 W/mm^2^ induced a 71.8% decay in the steady-state PL with respect to peak value (Figure 11a), as well as a non-recoverable 54.6% decrease in the reference PL signal (Figure 11b). Interestingly, the PL signal under high level of excitation shows a partial time-dependent recovery beginning after 1.5 h of stress. If we consider that thermally treated samples showed PL recovery during thermal treatment up to 300 °C, this behavior can be related to the thermally induced annealing of the material surrounding the excitation spot.

By comparing the experimental results outlined in this section, we can conclude that stress under high levels of optical excitation (i) triggers a very fast degradation process, which induces most of the non-recoverable PL decay during the first second of stress. Additionally, (ii) a recoverable PL decay is also present, which can be ascribed to the thermal quenching experienced by the luminescence material; (iii) below the critical excitation intensity, long-term exposition to optical excitation does not trigger any further degradation process.

The strong dependence of the onset of permanent PL decay on the excitation intensity also highlighted the major role of power dissipation, i.e., temperature, in the degradation process. From an engineering perspective, this means that while the maximum operating temperature of the material is an intrinsic characteristic of the phosphor, and thus can only be changed by improving either its composition or its manufacturing process, the excitation intensity threshold can be easily increased by improving the thermal management capabilities of the system. In particular, this goal can be achieved by lowering the thermal resistance from the phosphors grains to substrate, for example by incorporating the luminescent material in a highly thermally conductive encapsulant, or by increasing the thermal conductivity of the substrate, by making use of ceramic plates instead of the sapphire supports employed in this case of study.

## 5. Conclusions

In conclusion, with this work we investigated the robustness and the degradation mechanisms of blue-emitting ${\mathrm{Sr}}_{5}{{(\mathrm{PO}}_{4})}_{3}{\mathrm{Cl}\;:\;\mathrm{Eu}}^{2+}$ phosphors for near-UV laser excitation. By means of a series of long-term aging and short-term stress experiments under optical excitation and/or high-temperature environment, the limits for continuous operation of the luminescent material were found. In particular, for temperatures equal to or greater than 450 °C the material exhibited a time-dependent drop in the PL, which was attributed to the thermally induced auto-ionization of the Eu^2+^ optically active centers. By means of different material characterization techniques, evidence of this degradation process were also found on samples stressed under a relatively low 3.2 W/mm^2^ optical excitation density. This indicated that the optically (and thermally) induced ionization of the Eu^2+^ species is the most critical degradation process for this family of phosphorescent material. In addition to that, short-term stress under 405 nm optical excitation revealed that a threshold excitation intensity for continuous pumping of Eu-doped halophosphates luminescent pigments exists. The threshold value was found to be in the 1.5 W/mm^2^ to 2 W/mm^2^ range for the given deposition condition: this excitation threshold may depend on the specific morphology of the area under analysis, as well as on the thickness of the deposited material and on the type of substrate employed. Above threshold, decay of the steady-state PL intensity and/or degradation of the material occur with respect to lower excitation intensity, which suggests that the material is being operated in a not optimal excitation regime.
